# What does a breast feel like? A qualitative study among healthy women

**DOI:** 10.1186/s12905-018-0577-1

**Published:** 2018-06-01

**Authors:** Anouk J. M. Cornelissen, Stefania M. H. Tuinder, Esther M. Heuts, René R. W. J. van der Hulst, Jenny Slatman

**Affiliations:** 1 Department of Plastic Surgery, Maastricht University Medical Center, P. Debyelaan 25, 6229 HX Maastricht, The Netherlands; 2 Department of Surgery, Maastricht University Medical Center, P. Debyelaan 25, 6229 HX Maastricht, The Netherlands; 3 Department of Culture Studies, Tilburg School of Humanities and Digital Studies, Warandelaan 2, 5037 AB Tilburg, The Netherlands

**Keywords:** Breast, Sensation, Breast reconstruction, Qualitative study

## Abstract

**Background:**

Restoring the body as normal as possible increases quality of life. Aesthetically, almost perfect breast reconstructions can be created. However, these reconstructed breasts have almost no sensation. Our hypothesis is that if we succeed in restoring sensation, this will increase quality of life. So far, little is written about the phenomenon of breast sensation, which makes it difficult to evaluate whether the quality of life increases after restored sensation. Therefore, the primary goal of this study is to determine what the importance and meaning is of breast sensation among healthy women.

**Methods:**

A qualitative, descriptive phenomenological study was performed in an academic hospital between October 2016 and March 2017. A total of 10 semi-structured in-depth interviews were conducted in healthy women who did not undergo prior breast surgery. The sample size was based upon ‘saturation’. The interviews were tape-recorded, transcribed verbatim, coded and analysed according to phenomenology keeping in mind the research question ‘what is the importance and meaning of sensation of the breast?’

**Results:**

Seven interrelated themes on how sensation of the breast is experienced were found: the absent breast (1), the present breast (2), the well-functioning breast (2a), the feminine breast (2b), the sensual breast (2c), the alien breast (2d), the safe breast (2d).

**Conclusions:**

The seven interrelated themes can form the basis to develop a quantitative research tool to evaluate quality of life after innervated breast reconstruction and can be implemented in counselling before breast reconstructive surgery in the form of shared treatment decisions.

## Background

Quality of life after breast cancer treatment should be an important goal during treatment because breast cancer incidence and survival rate keeps growing due to changing lifestyle, early detection and advances in therapy [1]. Previous studies suggest that restoring the body as normal as possible after mastectomy, increases the quality of life [2–4]. Although excellent cosmetic results can be achieved with autologous breast reconstruction, most reconstructed breasts fail to regain sensation [5]. Technically, surgeons are able to perform a sensible nerve coaptation [6–10]. Currently, multiple randomized studies are on their way to provide level A evidence on the effect of nerve coaptation therefore it is not yet standard treatment.

Another reason why this technique is not yet widely spread might be that the (importance of) breast sensation is undervalued in literature. Some studies quantified the loss of sensation, measured by pressure on the skin, in women who underwent a breast operation [11–13]. However, breast sensation is far more complex than only pressure sensitivity, since the question ‘does your breast feel like your own’ seems to be one of the most important determinants of patient satisfaction after breast reconstruction [14].

Our hypothesis is that (the qualitative aspect of) breast sensation is important to women and if we can restore sensation of the reconstructed breast, quality of life of breast cancer survivors will improve further. This hypothesis could assume that techniques to improve sensation of the autologous reconstructed breast, should be encouraged [8]. However, to properly restore the sensation of the (reconstructed) breast we should, first, understand the phenomenon of breast sensation.

Therefore, the primary goal of this study is to explore the importance and meaning of sensation of the breast. The results can be used in a follow-up study in operated women, to finally develop an evaluation tool (questionnaire) to evaluate the qualitative aspect of breast sensation.

## Methods

### Study design

In the Maastricht University Medical centre a qualitative study, based on in-depth interviews was performed between October 2016 and March 2017. A phenomenological analysing method was used to address the research question ‘What is the importance and meaning of sensation of the breast?’. Phenomenology focusses on a person’s perception of an ‘event’ and tries to understand people’s perspective and understanding of a certain ‘phenomenon’ [15].

This study was approved by the local ethical committee (project 164,228). Written informed consent was retrieved from all participants.

### Participants

In total 10 interviews with healthy women who did not undergo prior breast surgery were performed. One interview was a duo interview with a couple. We used purposive sampling to interview highly educated women, to answer this research question subjects needed to be able to express themselves well. Previous research shows that age, body mass index (BMI) and breast size are inversely correlated to breast sensation [16, 17]. Therefore, we created heterogeneity for these factors. Characteristics of our participants can be found in Table 1. Participants were recruited through snowball sampling [18].

**Table 1:** Participants characteristics

#	Age	Ethnic identity (self-declared)	Sexuality	Children	Breastfeeding	Cup size	BMI	Education
1	25	Asian/Caucasian	Hetero	1	Yes	C	18	University
2	56	Caucasian	Hetero	1	Yes	B	22	University
3	74	Caucasian	Hetero	3	Yes	E	31	University
4	21	African	Hetero	0	No	A	30	Elementary
5	43	Caucasian	Hetero	2	Yes	B	25	University
6	20	Caucasian	Lesbian	0	No	A	23	University
7	21	Caucasian	Lesbian	0	No	A	20	University
8	22	Caucasian	Hetero	0	No	D	22	University
9	27	Caucasian	Hetero	0	No	D	21	University
10	25	Caucasian	Hetero	0	No	B	21	University

The sample size was calculated by ‘saturation’, so the appropriate sample size was met if interviews did not supply new themes [19].

### Semi-structured interviews

The in-depth interviews were semi-structured. A topic list was developed based upon brainstorming among the authors. (Table 2) This topic list was considered a dynamic instrument and changed over time based on new insights which developed as more interviews were performed. Participants were encouraged, by questions like ‘What do you mean?’ , ‘Could you give an example?’, to speak freely about their breasts. Interviews were held at the participants house or a location of choice e.g. a café. The interviews were tape-recorded, on average the interviews lasted 30 min.

**Table 2:** Topic list of themes of semi-structured in-depth interview

General information	Full name, age, BMI, cup size, children, breastfeeding, self-declared ethnic identity, sexuality and education.
Sensation of the breast	Breast self-exam, reaction of the nipple, pain, breastfeeding, development in life (child, puberty, adulthood and motherhood), erogenous zone
Menstrual cycle	Pain, difference in feeling, nipple
Hypothetical	No sensation in the breast, importance of sensation in reconstructed breast, situations in which sensation in the breast would be missed

### Analysis

The interviews were transcribed verbatim after which transcripts were anonymized for further analysis. Transcripts were first coded by hand, starting with descriptive open codes, like reaction of the nipple, progressing to clustering of these codes into axial codes, like sexuality and eventually themes [20]. Developing of these codes/themes was supported by the question ‘how do women experience sensation of the breast and what does it mean to them?’

We chose not to perform member checking. Since, this does not increase validity and might even pose a threat to validity if participants want to correct their answers. Furthermore, our goal is not internal validity but rather comprehensiveness of the phenomenon breast sensation [21]. To minimize the effect of subjectivity during analysis ‘Multiple Coding’ was performed in the form of supervised sessions during research meetings with the first and last author. Segments of the data with content of disagreement were coded multiple times and discussed to create further insight to refine our coding system [22].

The entire analysis was based upon a phenomenological approach, according to which we can explore how different meanings can be attributed to one’s body. Our analysis focuses on different meanings of the breast, i.e. the different ways in which the breast “appears” to a person.

## Results

Through the analysis of our data, we identified seven interrelated themes, which are derived from Leder’s distinction between *absent* and *present* body. (Table 3) These themes represent different ways in which sensation of the breast is related to different meanings of the breast in healthy women.

**Table 3:** Illustrating quotes of the seven identified themes of the phenomenon ‘breast sensation’ in healthy women with the respondents number

Theme		Quote
1 The absent breast	7	The thing is, off course I am not aware of my breasts, not even now.
	10	If you are asking me about these topics I am thinking ‘Oh yes I am aware of my breasts’. But in the moment, not so much, they are just always there … So, they have become part of my body and how my body works and reacts in certain environments.
	5	The fact that you are not aware (of your breast) is pleasant and will interfere with the way I describe sensation. Because they are just part of my body and I am not aware of them. It would be a pity (if I did not have sensation). But it is not something I would worry about. However, I am not sure. And off course it matters if sensation would be gone in one or both sides … I don’t think it would be such a big thing if I would still have sensation on one side.
2 The present breast	10	If I menstruate, I feel them a little bit more, it is an unpleasant sensation. If you don’t have your period you feel freer and don’t have to think about anything.
	9	I don’t find performing self-breast-exam annoying. I just feel neutral about it I just do it quickly, once a month Sometimes I feel pain, right before I have to menstruate, that is a very painful sensation in the breast.
2a The well-functioning breast	2	That that body can carry a child, can bear a child and can feed a child. I find that very pleasant. ‘Oh that is what these breasts are also for’.
2b The feminine breast	6	I would miss it (sensation in the breasts), but I don’t think… I would not feel less feminine… I think the aesthetic appearance of the breast are more important to feel feminine than sensation.
2c The sensual breast	3	Well, my partner thought it was amazing (breasts in an intimate setting). I thought, well if this is part of intimacy, well ok then. But me myself I did not find it special.
2d The alien breast	2	Sometimes I have to hold them while playing sports, and then it becomes almost a thing like during a mammography, a separate thing… That breast is pressed completely flat in between two plates and off course that is a very strange experience. A thing hanging from your body, I don’t like it at all… As if the breast is being taken away from you. I like to think of the breast as something round… That might nog be very realistic, if you see what kind of breasts there are. But the form changes (makes flat gestures with her hands) and that seems strange to me.
2e The safe breast	10	It might be scary (to have a breast without sensation) because I would think that if a body part is been operated you would be extra focussed on this body part and that you would want to know how it is doing. And if you don’t feel anything anymore, you would lose control… But I also think it (sensation of the breast) is important to regain trust in your body… For example, this might be a strange comparison, but I have injured my ankle once and during revalidation sensation was very important to see if I could move it and if it was going well with my ankle.
	7	It is not a pleasant sensation (sensitive breast during menstruation), but it is more like I know that my body is working right... You can trust a little bit on your body, I know by the sensation of my own breasts ok I will have my menstruation with a few days… It is a confirmation that everything is well, you know. And that is pleasant although the sensation is a bit painful.
	5	Because you know, for me the main reason to undergo a preventive mastectomy would be to get some security. And I think that if you cannot feel a piece, I think that, that would give me a very insecure feeling. There is a piece on my body, but I can’t feel it. For me that would be very unpleasant.

### The absent breast (1)

Most respondents were not aware of (sensation in) their breast during their daily life. The fact that their breasts were ‘absent’ was mentioned as a positive aspect. At the beginning, it was difficult to interview healthy women about their breast sensation which was not actively sensed. Moreover, our respondents said that sensation of the breast is an unfamiliar topic in conversations among friends.

During the interviews, hypothetical questions were asked like: ‘if you had no sensation in your breast, would you miss it?’. Paradoxically, all women would answer yes. Respondents who would choose for reconstructive breast surgery, if they ever needed to undergo a mastectomy, would choose for a reconstruction with sensation, if given the option. Respondents saw it as an extra advantage and mentioned it would be strange if somebody touched their breasts and they would not be able to register this, as normally this is an intimate place to be touched. Although, they found it very difficult to give specific examples of situations in which the lack of sensation would bother them. Mostly, the sensual sensation was mentioned to be missed, but also a feeling of security.

Some respondents answered that it would make an important difference whether only one or both breasts would have no sensation. They indicated that having one healthy breast with normal sensation would be ‘enough’ but if both breasts had no sensation they would be more eager to opt for a sensate reconstruction.

### The present breast (2)

The present breast could be present in many different ways to women. Therefore, the theme *present breast* was further subdivided into five themes; the well-functioning breast (2a), the feminine breast (2b), the sensual breast (2c), the alien breast (2d) and the safe breast (2e).

Instances of explicit breast sensation were mostly caused by ‘negative’ sensations like pain/unpleasant sensation. E.g. breasts become more sensitive/painful during menstruation, an unpleasant sensation while playing sports especially in women with larger breast, nipple reaction due to cold temperature etc. Women were also aware of their breast sensation during breast self-examination. However, respondents did not experience this in a negative nor positive way. One respondent described it as a business act, something that had to be done without any other associations.

Respondents claimed to have higher sensitivity and awareness in an intimate setting. This is discussed under the theme ‘the sensual breast’. One respondent even explained that when she wears a push-up bra with a deep cleavage top, whenever she goes out, she feels as if her breasts are ‘more present’, and she is more aware of her breast sensation.

### The well-functioning breast (2a)

If asked about breast sensation, all respondents who gave breastfeeding would spontaneously mention this. The sensation was described as a total different and new sensation of the breast (e.g. lactation, breast engorgement etc.). Although, for some breastfeeding was associated with unpleasant sensations of the breast e.g. nipple pain, fissures, painful breast engorgement, etc., they remembered it as a valuable experience and seemed to have forgotten the pain that came with it. Some women mentioned that being able to give breastfeeding would be an important factor if they had the choice about the timing of the prophylactic mastectomy, they would wait until after their family was complete.

Some women described the breast to be more sensitive in certain periods of the menstrual cycle, this was described as unpleasant but not necessarily painful. However, they preferred not to be touched on their breasts during this period.

The breast and especially the nipple-areola complex have another function, the nipple is considered an erogenous area. However, our respondents explained that touching of the nipples did not cause significant arousal and was not considered as an important erotic body part during sexual interaction.

### The feminine breast (2b)

Most respondents thought that having sensation or not in their breasts, would not influence their femininity. Whereas having breasts or not *was* thought to influence their femininity. Almost all respondents named restoring the appearance of a natural looking breast as the main reason to choose for a breast reconstruction. One respondent thought she would be able to feel feminine wearing clothes and external prosthesis, however she would not feel feminine in a bathing suit or naked after a mastectomy.

### The sensual breast (2c)

The nipple is considered an erogenous area and becomes erectile during arousal. Being touched there in an intimate setting was experienced as pleasant by our respondents. However, most respondents do not think, they would miss the contributing factor of the nipple to arousal. Most women had the idea that their male partner enjoyed touching the nipple more than they did. Most respondents did enjoy seeing their partner being aroused by their nipple.

### The alien breast (2d)

Some respondents explain that when the breast loses the normal round shape, they feel as if it is no longer a breast, but rather a ‘thing’ hanging from their body. Mammography was the most named source of this feeling. But also during sports women experienced their breast as not feeling like a breast.

### The safe breast (2e)

One respondent found it paradoxical that if she would choose for a risk-reducing bilateral mastectomy and her reconstructed breasts would have no sensation, she would not feel safer at all. However, this was the goal to undergo this operation to begin with. Another respondent compared it to having an injured ankle, to her it would be strange to have no sensation in an impaired body part, because normally sensation increases in an impaired body part and warns the body whenever there is a problem.

Some respondents, described hypersensitivity of their breast during certain periods in their menstrual cycle and experienced it as a confirmation that their body was still functioning ‘normal’ , although they experienced this sensation as unpleasant. For one respondent, this was the reason to change from an intrauterine hormonal device to oral contraceptives, in order to be able to have a menstrual cycle again.

## Discussion

Not much literature is written about sensation of the breast, the literature that *is* written focuses on ‘negative’ sensation (pain) [23–28]. This is to be expected according to the theory of Leder [29], generally, there is no awareness of healthy body parts, once body parts are impaired, one suddenly becomes aware of those body parts. This study shows that breasts mostly live an unnoted life, which is considered a positive thing. However, in our clinic after a breast reconstruction, patients will complain about their breasts not feeling like their own and paradoxically the breast(s) become(s) ‘*more present*’ although no breast sensation is present.

Our respondents stated that sensation was not needed to feel feminine, however a beautiful natural looking breast is, for our respondents this would be the main reason to choose for a reconstruction. This is noteworthy, since in reconstruction restoring function is normally the main goal. E.g. after leg amputation function can be restored by using running blades, which do not resemble a normal leg at all nor are aesthetically pleasing [30].

Remarkably, our respondents described a link between appearance and sensation; if a breast loses the round shape e.g. during a mammography, the breast is also sensed differently, like a ‘thing’. If the breasts are more present e.g. in a push-up bra, the breasts are sensed more actively. This emphasizes the importance of a natural looking breast after reconstruction also for the regain of sensation.

The goal of mastectomy in prophylactic cases is different compared to surgery after a malignancy. These women do not want to go from an insecurity because of possible malignancy in the future to a daily-based insecurity because of an absent breast, which paradoxically is more ‘*present’* because it becomes ‘impaired’ after surgery. To these women the information about sensation after the operation, might be even more contributing to the decision to undergo a prophylactic mastectomy.

As was stated by one of our responders she would not feel safer at all after a breast reconstruction without sensation. On the contrary, she would feel less safe if she would not have sensation in an ‘impaired’ breast. In women with a higher oncological risk, sensation might be even more important. However, further qualitative research in genetically predisposed women is necessary.

Interestingly, the erogenous sensation does not seem to play a major role in breast sensation according to our results. Our respondents indicated that mainly their partner enjoyed their breasts during an intimate setting, however for this function erogenous sensation is not necessary. This might be clinically relevant, because technically we are able to perform a microsurgical nerve coaptation and restore breast sensation, light touch [6]. However, restoration of erogenous sensation is rather an exception [10]. Previously, it was thought that nerve coaptation was not worthwhile if erogenous sensation could not be restored, this might be correlated to the number of female plastic surgeons, this used to be only 0.2% in 1959, now the number of female plastic surgical trainees is 37% [31], which might explain the renewed interest.

Another interesting fact, which might have clinical relevance is that some respondents indicate that having one breast with normal sensation would be sufficient. Currently, in unilateral breast reconstruction often the contralateral, healthy, breast is operated on to symmetrize the result. According to our results it might be important to discuss the implications for breast sensation of having a correction on the ‘healthy breast’ , in order to adapt the surgical plan according to your patient’s wishes.

This study was meant to find out what qualitative aspects of breast sensation are important and to lay the basis to develop a proper evaluation tool. Up until now, we can only rely on quantitative sensation measurements and existing questionnaires like the general questionnaires (SF-36, EORTC, Body image scale) and the disease specific questionnaire (BREAST-Q). However, none of these focus on (positive) sensation of the breast after breast cancer treatment. Developing a new tool specified on positive breast sensation, might improve the way of evaluating the results of innervated breast reconstruction and provide higher quality evidence, which is needed to be able to implement this technique in national guidelines.

We are aware of the fact that this study was performed among healthy women and these results cannot be extrapolated to women treated for breast cancer. This study was merely meant to provide a frame of reference about the phenomenon breast sensation in the form of seven interrelated themes which will serve as a base for a follow-up study in women who underwent breast surgery and to eventually develop a questionnaire to evaluate the impact on quality of life of innervated breast reconstructions.

## Conclusions

In this phenomenological qualitative study, seven interrelated themes that explain the different ways the breast can be ‘*absent*’ or ‘*present*’ in healthy women were found. This knowledge can be used to design new evaluation tools and can be implemented in counselling before breast reconstructive surgery in the form of shared treatment decisions.
